# Prognostic impact of prior percutaneous coronary intervention on patients undergoing coronary artery bypass grafting – A meta-analysis of reconstructed time-to-event data

**DOI:** 10.1016/j.ahjo.2025.100606

**Published:** 2025-09-13

**Authors:** Hristo Kirov, Tulio Caldonazo, Hermann Woehlecke, Luca Fazzini, Johannes Fischer, Vlander Costa, Paulo Amorim, Angelique Runkel, Eduardo Rodrigues, Murat Mukharyamov, Mauro P.L. de Sá, Torsten Doenst

**Affiliations:** a Department of Cardiothoracic Surgery, Friedrich-Schiller-University Jena, Germany; b Department of Medical Sciences and Public Health, Clinical Cardiology Unit, University of Cagliari, Cagliari, Italy; c Department of Cardiovascular Medicine, Mayo Clinic, Rochester, United States; d Federal University of Rio de Janeiro, Rio de Janeiro, Brazil

**Keywords:** Percutaneous coronary intervention, Coronary artery bypass grafting, Left main coronary disease

## Abstract

**Background:**

There is controversy on the effect of percutaneous coronary intervention (PCI) on outcomes of patients undergoing coronary artery bypass grafting (CABG). We meta-analytically assessed the prognostic impact of prior PCI in patients with coronary artery disease (CAD) who underwent CABG.

**Methods:**

We performed a systematic review and meta-analysis of studies comparing patients who underwent CABG and had prior PCI in the past with patients who underwent CABG as primary treatment of CAD. Three databases were assessed. The primary endpoint was perioperative mortality. The secondary outcomes were long-term survival, perioperative myocardial infarction, neurological events, bleeding, acute renal failure, and hospital length of stay. Reconstruction of time-to-event data and pairwise meta-analysis were performed.

**Results:**

Nineteen studies met the criteria for inclusion in the final analysis. Risk of perioperative mortality in patients undergoing CABG after a prior PCI was higher than in those undergoing primary CABG (OR: 1.16, 95 % CI, 1.03–1.31, *p* = 0.02). However, the prior PCI group presented higher survival rates when compared to the primary CABG group over the entire follow-up (HR: 0.90, 95 % CI, 0.86–0.94, *p* < 0.01). There was no significant difference between the groups regarding the other secondary outcomes.

**Conclusions:**

When compared with patients who underwent CABG as primary treatment of CAD, prior PCI is associated with higher perioperative mortality for patients undergoing CABG. However, this increase in perioperative risk does not correlate with a decrease in long-term survival.

## 1 Introduction

Coronary artery disease (CAD) is one of the leading causes of mortality and morbidity worldwide among cardiovascular diseases (1,2). Most patients with CAD undergo invasive coronary interventions (3,4). The main invasive strategies are percutaneous coronary intervention (PCI) and coronary artery bypass graft surgery (CABG) (5). Over the past twenty years, PCI has become the predominant therapy in Western world (6).

Recently, extensive research and advancements in PCI techniques and devices, including new generations of drug-eluting stents, have enabled interventionalists to treat more complex CAD in older and sicker patients with PCI (7). Additionally, patients presenting with ST-segment elevation acute coronary syndrome are most often treated with PCI, regardless of coronary anatomy complexity, as emergency CABG is infrequently performed (8). Consequently, there is an increasing number of patients who underwent previous PCI and later require CABG, which increases the surgical complexity of the case and makes CABG more challenging (9,10).

For instance, a prior PCI has been suggested as an independent risk factor for in-hospital mortality after CABG (11,12). However, whether these patients have a worse prognosis in the long-term, remains elusive.

We performed a systematic review and meta-analysis of studies that compared patients undergoing CABG with a prior PCI to those who underwent CABG as the primary treatment for CAD.

## 2 Methods

Ethical approval of this analysis was not required as no human or animal subjects were involved. This review was registered with the National Institute for Health Research International Registry of Systematic Reviews (PROSPERO, CRD42023452791).

### 2.1 Search strategy

We performed a comprehensive literature search to identify contemporary studies reporting a comparison between patients who underwent CABG and had prior PCI in the past with patients who underwent CABG as the primary treatment of CAD. Searches were run on August 2023 in the following databases: Ovid MEDLINE; Scopus and Web of Science. The search strategy is available in Supplementary Table 1.

### 2.2 Study selection

The study selection followed the Preferred Reporting Items for Systematic Reviews and Meta-Analyses (PRISMA) strategy. After de-duplication, records were screened by two independent reviewers (HW and AR). Any discrepancies and disagreements were resolved by a third author (HK). Titles and abstracts were reviewed against pre-defined inclusion and exclusion criteria.

### 2.3 Eligibility criteria

Studies were considered for inclusion if they reported direct comparison of outcomes between populations undergoing CABG with or without prior PCI. The inclusion also required the availability of the reported outcomes of interest.

Exclusion criteria were studies lacking outcomes of interest, conference abstracts and proceedings, case reports, and non-comparative study designs. The full text was pulled for a second round of eligibility screening. References of the selected articles were also reviewed for relevant studies not captured by the original search. The quality of the included studies was assessed using the Newcastle-Ottawa Scale (Supplementary Table 2).

Two reviewers (HW and AR) independently performed data extraction. Accuracy was verified by a third author (HK). The extracted variables included study characteristics (publication year, country, sample size, study design, and selected outcomes) as well as patient demographics (age, sex, body mass index – BMI, hypertension - HP, diabetes mellitus - DM, dyslipidemia, smoking status, mean left ventricular ejection fraction – LVEF, prior myocardial infarction - MI, atrial fibrillation – AF, chronic obstructive pulmonary disease – COPD, cerebrovascular accident and peripheral vascular disease – PVD).

### 2.4 Outcomes

The primary outcome was perioperative mortality. Secondary outcomes were long-term survival, perioperative myocardial infarction (MI), neurological events (NE), bleeding, acute renal failure (ARF), and hospital length of stay (LOS).

### 2.5 Statistical analysis

We conducted meta-analyses to compare the outcomes of patients undergoing CABG with and without prior PCI. Odds Ratios (OR) and 95 % confidence intervals (CI) were calculated for the short-term outcomes. The results are displayed in forest plots. An OR greater than one indicates that the outcome is more frequently present in the prior PCI arm. Inherent clinical heterogeneity between the studies was balanced via the implementation of a random effects model. Between-study statistical heterogeneity was assessed with the Cochran Q statistic and by estimating I^2^. High heterogeneity was confirmed with a significance level of *p* < 0.10 and I^2^ of at least 50 % or more. We used a reconstructed time-to-event data strategy for the long-term survival (13,14).

### 2.6 Individual patient survival data meta-analysis

We used the methods described by Wei et al. to reconstruct individual patient data (IPD) from the Kaplan-Meier curves of all eligible studies for the long-term outcome (13,14). Raster and Vector images of the Kaplan–Meier survival curves were pre-processed and digitized, so that the values reflecting to specific timepoints with their corresponding survival/mortality information could be extracted. Where additional information (e.g., number-at-risk tables or total number of events) were available, they were used to further calibrate the accuracy of the time-to-events. To confirm the quality of the timing of failure events captured, we thoroughly checked the consistency with the reported survival or morality data provided in the original publications.

### 2.7 Meta-analysis of reconstructed data

The Kaplan–Meier method was used to calculate the overall long-term survival. The Cox proportional hazards regression model was used to assess between-group differences. For these Cox models, the proportional hazards assumption was verified by plotting scaled Schoenfeld residuals and log–log survival plots. We plotted survival curves using the Kaplan–Meier product limit method and calculated the HRs and 95 % CIs of each group. A HR greater than 1 indicated lower survival rates in the prior PCI arm. All statistical analyses were performed using R (version 4.3.1, R Project for Statistical Computing) within RStudio and STATA IC17.0 (StataCorp LLC, College Station, Texas).

## 3 Results

### 3.1 Study characteristics

**Fig. 1:**
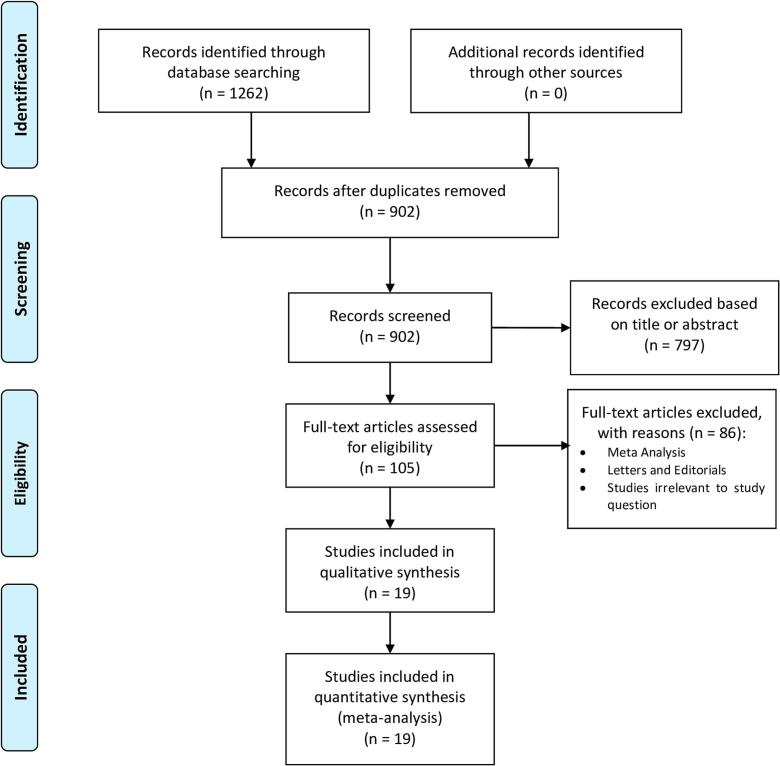
Preferred reporting items for systematic reviews and meta-analyses (PRISMA) flow diagram.

A total of 902 studies were retrieved from the systematic search, of which 19 met the criteria for inclusion in the final analysis. Fig. 1 shows the PRISMA flowchart for study selection. The included studies were published between 2002 and 2023.

Table 1 shows the details of the included studies. A total of 116,306 patients were included in the final analysis. The number of patients in each study ranged from 200 to 34,316.

**Table 1:** Baseline characteristics of included studies.

Author	Year of publication	Country	N° of patients	Study design	Reported endpoint
Barakate (26)	2002	Australia	12,270	Retrospective	Perioperative mortality, MI, NE, bleeding, ARF, and hospital LOS
Biancari (27)	2018	Europe	6563	Prospective, multicenter registry	Perioperative mortality, NE, bleeding, ARF
Biancari (28)	2022	Europe	2619	Prospective, multicenter registry	Long-term survival
Cheng (29)	2016	Taiwan	439	Retrospective	Perioperative mortality, long-term survival, NE, bleeding, ARF, and hospital LOS
Eifert (30)	2010	Germany	200	Prospective	Perioperative mortality, long-term survival, MI, ARF, and hospital LOS
Hakamada (31)	2021	Japan	1651	Retrospective	Perioperative mortality, long-term survival, NE, bleeding
Hamiko (16)	2023	Germany	748	Retrospective	Perioperative mortality, long-term survival, NE, and hospital LOS
Luthra (32)	2016	United Kingdom	660	Retrospective	Perioperative mortality, long-term survival
Mannacio (33)	2012	Italy	1704	Retrospective, multicenter	Perioperative mortality, long-term survival, MACE
Massoudy (34)	2009	Germany	29,928	Retrospective, multicenter	Perioperative mortality, MACE
Mehta (35)	2012	United States	34,316	Retrospective, multicenter	Perioperative mortality, MI, NE, bleeding, ARF, and hospital LOS
Miguel (36)	2019	Portugal	522	Retrospective	Perioperative mortality, long-term survival, MI, NE, MACE
Nardi (37)	2022	Italy	938	Retrospective	Perioperative mortality, long-term survival, NE, bleeding, ARF
Niclauss (38)	2015	France	1669	Retrospective	Perioperative mortality, MI, NE, bleeding, and hospital LOS
O'Neil (39)	2013	United States	13,354	Retrospective	Long-term survival
Rai (40)	2020	United Kingdom	2116	Retrospective	Perioperative mortality, long-term survival, NE, and hospital LOS
Stevens (41)	2009	United States	3236	Retrospective	Perioperative mortality, long-term survival, MI, NE, bleeding, ARF, and hospital LOS
Thielmann (17)	2021	Germany	2423	Retrospective	MI, NE
Velicki (42)	2013	Serbia	950	Retrospective	Perioperative mortality, hospital LOS, MACE

ARF: acute renal failure; LOS: length of stay; MACE: major adverse cardiovascular events; MI: myocardial infarction; NE: neurological events.

### 3.2 Patient characteristics

Supplementary Table 3 summarizes the demographic data of the patient population in each study. Age ranged from 61 to 69 years. Percentage of male patients ranged from 71 to 86 %; mean BMI ranged from 26.4 to 30.1 kg/m2; percentage of HP ranged from 39 to 89 %; percentage of DM ranged from 15 to 58 %; percentage of dyslipidemia ranged from 40 to 91 %; percentage of current smokers ranged from 10 to 67 %; mean LVEF ranged from 49.9 to 65.2 %; percentage of prior MI ranged from 17 to 77 %; percentage of COPD ranged from 4 to 27 %; percentage of prior CVA ranged from 2 to 17 %; and percentage of PVD ranged from 4 to 24 %.

### 3.3 Primary outcome

**Fig. 2:**
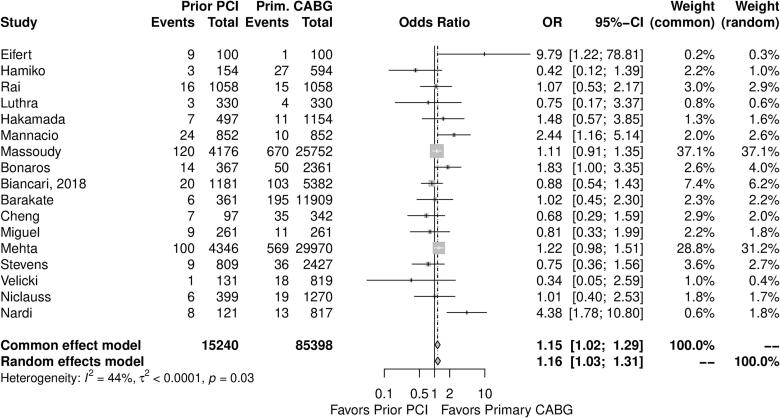
Forest plot for the primary endpoint perioperative mortality. CABG = coronary artery bypass grafting; CI = confidence interval; PCI = percutaneous coronary intervention; OR = odds risk.

Table 2 provides a summary of outcomes from the included studies. Fig. 2 shows the forest plot for perioperative mortality, which was assessed in 17 studies comprising 100,638 patients. Compared to patients who underwent prior CABG, the prior PCI group was associated with higher perioperative mortality (OR: 1.16, 95 % CI, 1.03–1.31, *p* = 0.02).

**Table 2:** Summary of outcomes.

Outcome	Number of studies	Number of patients	Effect estimate, (95 % CI, *p*-value)
Perioperative mortality	17	100,638	OR: 1.16, 95 % CI, 1.03–1.31, *p* = 0.02
Long-term survival	12	30,661	HR: 0.90, 95 % CI, 0.86–0.94, *p* < 0.01
Myocardial infarction	7	54,636	OR: 1.20, 95 % CI, 0.89–1.62, *p* = 0.24
Neurological events	12	66,778	OR: 1.03, 95 % CI, 0.87–1.23, p = 0.71
Bleeding	8	61,064	OR: 1.26, 95 % CI, 0.95–1.66, *p* = 0.10
Acute renal failure	7	57,962	OR: 1.21, 95 % CI, 0.86–1.69, p = 0.27
Hospital length of stay	9	56,135	SMD: −0.26, 95 % CI, −0.70 to 0.18, p = 0.24

CI: confidence interval, HR: hazard ratio, SMD: standard mean difference, OR: odds ratio.

The leave-one-out analysis was consistent with the main analysis (Supplementary Fig. 1). Supplementary Fig. 2 shows the funnel plot from the perioperative mortality outcome. No evidence suggestive of publication bias was observed (*p* = 0.93).

### 3.4 Long-term survival

Overall, 12 Kaplan-Meier curves were processed, digitalized, and reconstructed. Using the previously described methodology, we extracted the IPD from these curves. The entire observation period was 19 years.

**Fig. 3:**
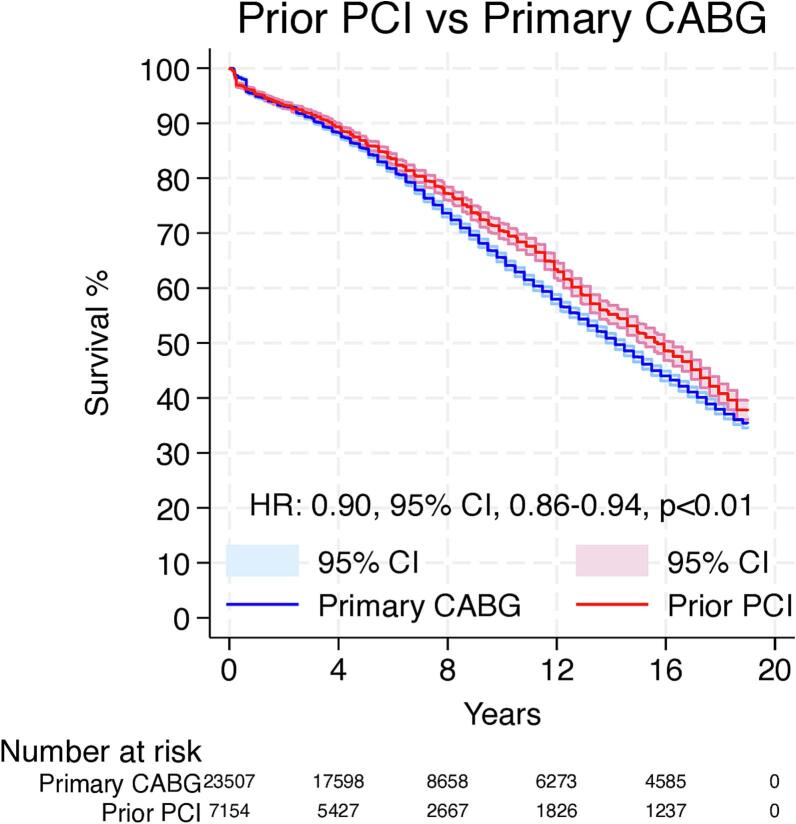
Pooled survival curve for the entire follow-up. CABG = coronary artery bypass grafting; CI = confidence interval; HR = hazard ratio; PCI = percutaneous coronary intervention.

Fig. 3 shows the pooled Kaplan-Meier curves for the entire observation period for long-term survival. The patients who underwent CABG as a primary treatment showed lower long-term survival when compared to the prior PCI group (HR: 0.90, 95 % CI, 0.86–0.94, *p* < 0.01).

### 3.5 Landmark analysis

Violation of the proportional hazards assumption was observed between scaled Schoenfeld residuals and follow-up time, as well as in log-log survival plots, which indicates that the HR is not constant over time (Supplementary Fig. 3).

**Fig. 4:**
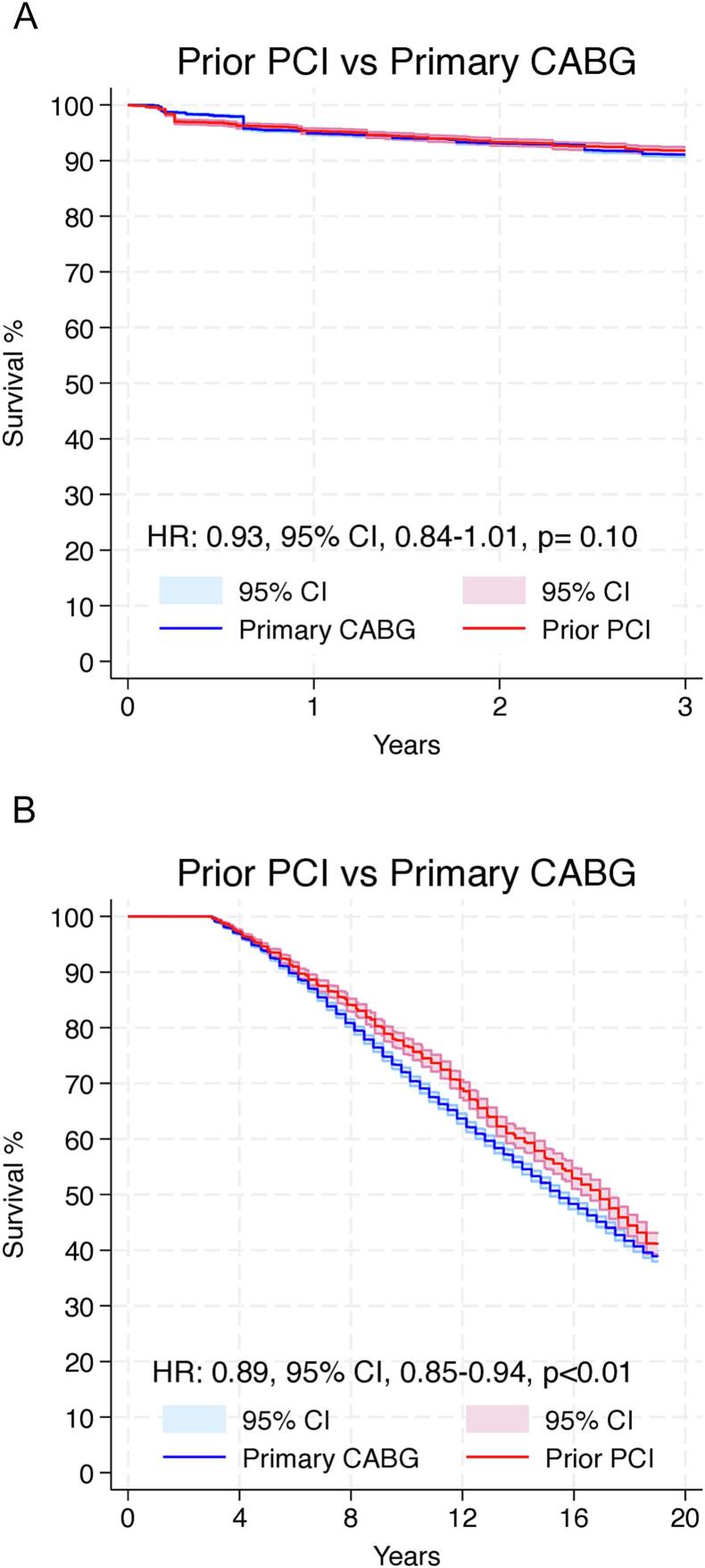
Landmark analysis comparing the survival rates during the first 3 years (A) and from 3 to 19 years (B). CABG = coronary artery bypass grafting; CI = confidence interval; HR = hazard ratio; PCI = percutaneous coronary intervention.

Since we observed that the proportional hazards assumption was violated, we proceeded with landmark analysis, designating 3 years as the landmark timepoint. Fig. 4A shows the 3 years survival analysis, which suggested no difference between the groups (HR: 0.93, 95 % CI, 0.84–1.01, *p* = 0.10). Fig. 4B shows the landmark analysis from 3 to 19 years, which suggested that compared to patients who underwent prior CABG, the prior PCI group was associated with higher survival rates (HR: 0.89, 95 % CI, 0.85–0.94, *p* < 0.01).

### 3.6 Secondary outcomes

There was no significant difference in the other secondary outcomes among groups. MI was evaluated in 7 studies with 54,636 patients (OR: 1.20, 95 % CI, 0.89–1.62, *p* = 0.24 - Supplementary Fig. 4), NE were assessed in 12 studies with 66,778 patients (OR: 1.03, 95 % CI, 0.87–1.23, *p* = 0.71 - Supplementary Fig. 5), bleeding in 8 studies including 61,064 patients (OR: 1.26, 95 % CI, 0.95–1.66, *p* = 0.10 - Supplementary Fig. 6), ARF in 7 studies including 57,962 patients (OR: 1.21, 95 % CI, 0.86–1.69, *p* = 0.27 - Supplementary Fig. 7), hospital LOS in 9 studies with 56,135 patients (SMD: −0.26, 95 % CI, −0.70 to 0.18, *p* = 0.24 - Supplementary Fig. 8).

## 4 Discussion

We demonstrate in this meta-analysis that prior PCI is associated with higher perioperative mortality for patients undergoing CABG compared with patients who underwent CABG as primary treatment of CAD. However, this increase in perioperative risk does not correlate with a decrease in long-term survival.

We found that [1] patients with a history of PCI who underwent CABG, had higher perioperative mortality; [2] 3-year survival was similar between groups, while [3] the long-term survival was higher in patients with a history of prior PCI; [4] we did not find any differences in secondary outcomes including MI, NE, bleeding, ARF, and hospital LOS.

Although our results may seem unexpected, there are several aspects that might explain them and require further discussion.

As PCI has become widely accessible, the profile of patients undergoing CABG has also evolved (15). Accordingly, nowadays, CABG candidates tend to be older with more comorbidities, and many have previously undergone PCI. The studies we included in our systematic review reported the proportion of such higher risk patients between 10 % and 30 % (16). We observed an overall higher risk of perioperative mortality among patients with a prior history of PCI. Several factors may contribute to the poorer outcomes observed in this subgroup. However, underlying mechanisms can only be hypothesized.

Although we might assume that the prior PCI group has severe CAD, the group without prior PCI might exhibit even more severe CAD, explaining their upfront referral for CABG.

A possible reason, explaining the higher perioperative mortality might be explained by the possible referral to CABG in acute coronary syndrome patients after failed

PCI. Some studies have demonstrated that acute or subsequent CABG within 24 h of PCI, as well as unsuccessful PCI in this context, is linked to a substantial perioperative risk and increased rates of cardiogenic shock (17). This association correlates strongly with heightened morbidity and mortality following CABG (17).

It is worth noting, however, that CABG is also performed in ACS cases following successful stenting of the culprit lesion. The timing of these procedures has evolved over time, influencing the risk profile for CABG, with variations in risk depending on the specific timing and clinical scenario.

A surprising finding from our analysis is that survival is similar in both groups up to 3 years but survival curves diverge thereafter, showing a better survival in the prior PCI group. A plausible explanation might be delivered by the fact that CABG patients are generally less likely than patients who have received PCI to adhere to secondary preventive medications (18). Thus one might speculate that the adherence to optimal medical therapy was better in the prior PCI group. Such assumption would in fact be able to explain the long-term survival difference observed in our study, as it has been showed that even the suboptimal adherence to medical therapy might affect long term outcomes, irrespective of the suboptimal medication usage (i.e. aspirin, β-Blocker or lipid-lowering therapy) (19). This has been confirmed by other studies showing that, patients undergoing CABG with optimal medical therapy had lower 10-year mortality than those without it (20). Moreover, it has been shown that medical therapy (i.e., dual antiplatelet therapy) may positively impact graft patency, further contributing to improved long-term outcomes (21).

This finding of better survival in the previous PCI group might be partially explained also by the hypothesis that patients who underwent primarily CABG present a higher CAD burden which might probably reflect also a higher systemic atherosclerotic burden ([22], [23], [24], [25]). Importantly, other factors related to survival outcomes such as a left ventricular function revealed similar values of ejection fraction measurements among the studies that reported the ejection fraction measurements in both groups.

The finding of a higher long-term mortality risk in patients without prior PCI compared with those who had undergone PCI before CABG suggests that, despite the observed increase in perioperative mortality in the prior PCI group, the long-term prognostic trajectory may be more favorable for these patients. This could reflect a selection effect, whereby patients surviving the early perioperative period after CABG in the context of prior PCI represent a lower-risk or better-optimized cohort, potentially due to more intensive medical management, closer follow-up, or the myocardial protective effects of staged invasive therapy. Alternatively, the absence of prior PCI in patients undergoing primary CABG may be a surrogate for more advanced or diffuse coronary disease at presentation, limiting the durability of surgical treatment.

The observed heterogeneity across outcomes may be partly explained by variations in study design, patient populations, surgical techniques, and endpoint definitions.

For instance, for perioperative mortality, heterogeneity is likely driven by differences in perioperative management protocols, the timing between PCI and CABG, and baseline patient risk profiles such as urgency of surgery and comorbidity burden. In the case of myocardial infarction, inconsistent definitions — ranging from perioperative biomarker-based criteria to clinical diagnoses — and varying sensitivity of postoperative surveillance may have contributed to divergent results. For bleeding, variability may stem from the use of different classification systems (e.g., TIMI, BARC, or institutional criteria), perioperative antithrombotic regimens, and surgical hemostatic practices. Acute renal failure outcomes are influenced by heterogeneity in creatinine-based definitions, thresholds for initiating dialysis, and baseline renal function across studies. Finally, differences in hospital length of stay may reflect institutional discharge policies, variations in healthcare systems, and the case-mix of elective versus urgent procedures.

### 4.1 Study strengths and limitations

This is the first meta-analysis of reconstructed time-to-event data to address this important topic. Besides perioperative mortality, six other endpoints were addressed in depth. Moreover, sensitivity analyses were performed for the primary endpoint and for the long-term data. However, this work has the intrinsic limitations of observational series, including the risk of methodological heterogeneity of the included studies and residual confounders. No adjustment for confounders was performed at the meta-analysis level, as this was a study-level rather than a patient-level analysis, and therefore post hoc adjustments are not possible by definition. Nevertheless, the majority of the included studies implemented baseline risk-adjustment methods, such as propensity score matching or multivariable regression, which partially mitigates the impact of confounding on the pooled estimates.

## 5 Conclusion

When compared with patients who underwent CABG as the primary treatment for CAD, those with a history of prior PCI experienced higher perioperative mortality. Interestingly, despite this elevated short-term risk, prior PCI did not translate into worse long-term survival. These results underscore the importance of careful perioperative management and risk stratification in patients with prior PCI undergoing CABG, while also highlighting that prior PCI should not be considered a deterrent to surgical treatment when clinically indicated.

## CRediT authorship contribution statement

**Hristo Kirov:** Writing – review & editing, Writing – original draft, Validation, Resources, Methodology, Investigation, Formal analysis, Conceptualization. **Tulio Caldonazo:** Writing – review & editing, Writing – original draft, Visualization, Validation, Software, Resources, Methodology, Investigation, Funding acquisition, Formal analysis, Data curation, Conceptualization. **Hermann Woehlecke:** Funding acquisition, Formal analysis, Data curation, Conceptualization. **Luca Fazzini:** Resources, Methodology, Investigation. **Johannes Fischer:** Methodology, Investigation, Funding acquisition, Formal analysis. **Vlander Costa:** Visualization, Validation, Investigation. **Paulo Amorim:** Methodology, Funding acquisition, Conceptualization. **Angelique Runkel:** Software, Resources, Project administration, Investigation, Formal analysis. **Eduardo Rodrigues:** Visualization, Validation, Supervision, Methodology, Investigation. **Murat Mukharyamov:** Supervision, Project administration, Formal analysis, Data curation, Conceptualization. **Mauro P.L. de Sá:** Visualization, Supervision, Resources, Investigation. **Torsten Doenst:** Writing – review & editing, Writing – original draft, Visualization, Validation, Supervision, Resources, Project administration, Methodology, Funding acquisition, Conceptualization.

## Funding

TC was funded by the 10.13039/501100001659Deutsche Forschungsgemeinschaft (DFG, German Research Foundation) Clinician Scientist Program OrganAge funding number 413668513, by the 10.13039/501100005971Deutsche Herzstiftung (DHS, German Heart Foundation) funding number S/03/23 and by the Interdisciplinary Center of Clinical Research of the Medical Faculty Jena.

## Declaration of competing interest

The authors declare that they have no known competing financial interests or personal relationships that could have appeared to influence the work reported in this paper.

## Data Availability

The data underlying this article are available in the article and in its online supplementary material.

## Appendix A Supplementary data

Supplementary material Image 1
