# Supplementary material for: Prognostic impact of prior percutaneous coronary intervention on patients undergoing coronary artery bypass grafting – A meta-analysis of reconstructed time-to-event data
Source: Am Heart J Plus. 2025 Sep 13;59:100606. doi: 10.1016/j.ahjo.2025.100606 (PMC12465058; doi:10.1016/j.ahjo.2025.100606)
Supplement: Supplementary file 1 — Supplementary material [file mmc1.docx]

**Supplementary Material**

**Supplementary Table 1.** Search strategy for Ovid MEDLINE.

**Supplementary Table 2**. Assessment of risk of bias using the Newcastle Ottawa Scale.

**Supplementary Table 3.** Demographics of included patients from the selected studies.

**Supplementary Figure 1.** Leave-one-out analysis for the primary endpoint (perioperative mortality).

**Supplementary Figure 2.** Funnel plot for the primary endpoint (perioperative mortality).

**Supplementary Figure 3.** Test of proportional hazard assumption (A) and in log–log survival plots (B).

**Supplementary Figure 4.** Forest plot for myocardial infarction.

**Supplementary Figure 5.** Forest plot for neurological events.

**Supplementary Figure 6.** Forest plot for bleeding.

**Supplementary Figure 7.** Forest plot for acute renal failure.

**Supplementary Figure 8.** Forest plot for hospital length of stay.

**Supplementary References**

**Supplementary Table 1.** Search strategy for Ovid MEDLINE (A) and Scopus/Web of Science (B).

A

| (“mortality"[MeSH Terms] OR "mortality"[All Fields] OR "mortalities"[All Fields] OR "mortality"[MeSH Subheading]) AND ("prior"[All Fields] OR "priors"[All Fields]) AND ("percutaneous coronary intervention"[MeSH Terms] OR ("percutaneous"[All Fields] AND "coronary"[All Fields] AND "intervention"[All Fields]) OR "percutaneous coronary intervention"[All Fields]) AND ("coronary artery bypass"[MeSH Terms] OR ("coronary"[All Fields] AND "artery"[All Fields] AND "bypass"[All Fields]) OR "coronary artery bypass"[All Fields] OR ("coronary"[All Fields] AND "artery"[All Fields] AND "bypass"[All Fields] AND "grafting"[All Fields]) OR "coronary artery bypass grafting"[All Fields]) |
| --- |
| **Translations** |
| **Mortality:** "mortality"[MeSH Terms] OR "mortality"[All Fields] OR "mortalities"[All Fields] OR "mortality"[Subheading]  **Prior:** "prior"[All Fields] OR "priors"[All Fields]  **Percutaneous coronary intervention:** "percutaneous coronary intervention"[MeSH Terms] OR ("percutaneous"[All Fields] AND "coronary"[All Fields] AND "intervention"[All Fields]) OR "percutaneous coronary intervention"[All Fields]  **coronary artery bypass grafting:** "coronary artery bypass"[MeSH Terms] OR ("coronary"[All Fields] AND "artery"[All Fields] AND "bypass"[All Fields]) OR "coronary artery bypass"[All Fields] OR ("coronary"[All Fields] AND "artery"[All Fields] AND "bypass"[All Fields] AND "grafting"[All Fields]) OR "coronary artery bypass grafting"[All Fields] |

B

| (mortality) AND (prior percutaneous coronary intervention) AND (CABG) |
| --- |

**Supplementary Table 2**. Assessment of risk of bias using the Newcastle Ottawa Scale.

| STUDY | SELECTION | COMPARABILITY | OUTCOME/ EXPOSURE |
| --- | --- | --- | --- |
| Barakate, 2002 (1) | **** | * | *** |
| Biancari, 2018 (2) | **** | ** | *** |
| Biancari, 2022 (3) | **** | ** | *** |
| Cheng, 2016 (4) | **** | ** | *** |
| Eifert, 2010 (5) | **** | ** | *** |
| Hakamada, 2021(6) | **** | ** | *** |
| Hamiko, 2023 (7) | **** | * | ** |
| Luthra, 2016 (8) | **** | ** | *** |
| Mannacio, 2012 (9) | **** | ** | *** |
| Massoudy, 2009 (10) | **** | ** | *** |
| Mehta, 2012 (11) | **** | ** | ** |
| Miguel, 2019 (12) | **** | ** | *** |
| Nardi, 2022 (13) | **** | * | ** |
| Niclauss, 2015 (14) | **** | ** | *** |
| O’Neil, 2013 (15) | **** | ** | *** |
| Rai, 2020 (16) | **** | ** | *** |
| Stevens, 2009 (17) | **** | ** | *** |
| Thielmann, 2021(18) | **** | ** | *** |
| Velicki, 2013 (19) | **** | * | *** |

**Supplementary Table 3.** Demographics of included patients from the selected studies (part 1).

| Study | Age  (mean±SD) | | Male  (%) | | BMI (kg/m^2^)  (mean ± SD) | | HP  (%) | | DM  (%) | | Dyslipidemia  (%) | | Smoking  (%) | | LVEF (%)  (mean ± SD) | |
| --- | --- | --- | --- | --- | --- | --- | --- | --- | --- | --- | --- | --- | --- | --- | --- | --- |
|  | PCABG | PPCI | PCABG | PPCI | PCABG | PPCI | PCABG | PPCI | PCABG | PPCI | PCABG | PPCI | PCABG | PPCI | PCABG | PPCI |
| Barakate, 2002 (1) | 61.6 | 60.0 | 80 | 75 | - | - | 44 | 39 | 15 | 17 | 52 | 48 | 67 | 60 | 56 | 65 |
| Biancari, 2018 (2) | 67.8±9.3 | 66.2±9.6 | 83 | 86 | 27.5±4.1 | 27.5±4.2 | - | - | 30 | 32 | - | - | - | - | - | - |
| Biancari, 2022 (3) | 67.6±9.2 | 65.6±10.0 | 83 | 84 | - | - | - | - | 29 | 32 | - | - | - | - | - | - |
| Cheng, 2016 (4) | 63.2 ± 0.6 | 65.3 ± 1.1 | 81 | 77 | - | - | 80 | 79 | 52 | 58 | - | - | 58 | 59 | - | - |
| Eifert, 2010 (5) | 65,9 ± 9,9 | 63 ± 10,5 | 80 | 80 | 27,2±3,9 | 26,38±3,87 | 83 | 88 | 26 | 31 | 84 | 85 | - | - | 60,47±14,1 | 60,04±14,5 |
| Hakamada, 2021 (6) | 69 ± 9,3 | 69 ± 9,7 | 75 | 79 | - | - | 80 | 82 | 53 | 54 | 68 | 73 | 20 | 17 | - | - |
| Hamiko, 2023 (7) | 67.9± 9.2 | 66.9± 9.2 | 81 | 81 | - | - | 76 | 76 | 40 | 45 | 62 | 64 | 30 | 29 | - | - |
| Luthra, 2016 (8) | 65.6 ±9.5 | 63.1±9.9 | 84 | 83 | 28.2 ±4.5 | 28.6±5.2 | 69 | 59 | 29 | 25 | - | - | 13 | 10 | - | - |
| Mannacio, 2012 (9) | - | - | 75 | 72 | - | - | 56 | 52 | 38 | 34 | 48 | 46 | - | - | - | - |
| Massoudy, 2009 (10) | 66.4 ±9.2 | 65 ±9 | 73 | 74 | - | - | 84 | 89 | 28 | 29 | 75 | 82 | - | - | - | - |
| Mehta, 2012 (11) | 64.1±10.7 | 62.8±10.7 | 73 | 75 | 30.1±24.2 | 30.13±11.1 | 77 | 84 | 38 | 41 | 76 | 87 | 54 | 53 | 51.5±12.6 | 50.26±12.0 |
| Miguel, 2019 (12) | 61.3±9.9 | 61.0±10 | 72 | 74 | - | - | - | - | 35 | 41 | 40 | 54 | 18 | 12 | 65.2±13 | 63.3±12.7 |
| Nardi, 2022 (13) | - | - | - | - | - | - | - |  | 31 | 49 | - | - | - | - | 53 ± 8.6 | 50 ± 9.6 |
| Niclauss, 2015 (14) | 67±10 | 66±10 | 81 | 81 | - | - | 71 | 75 | 32 | 35 | 72 | 67 | 46 | 46 | - | - |
| O’Neil, 2013 (15) | 64±10 | 62±10 | 71 | 70 | 29±5.5 | 30±5.8 | 72 | 76 | 35 | 36 | - | - | 24 | 25 | - | - |
| Rai, 2020 (16) | 63 (56-71) | 64 (56-71) | 81 | 81 | 27.9 | 28.0 | 73 | 73 | - | - | - | - | 11 | 11 | - | - |
| Stevens, 2009 (17) | 64±10 | 63±11 | 71 | 70 | - | - | 83 | 82 | 43 | 43 | 91 | 90 | 15 | 14 | - | - |
| Thielmann, 2021 (18) | 68±11 | 67±10 | 79 | 76 | 27.9±4.7 | 28.1±4.9 | 83 | 89 | 31 | 33 | 53 | 65 | - | - | 50.5±14.7 | 49.9±14.5 |
| Velicki, 2013 (19) | 62.4±9.3 | 60.6±9.2 | 72 | 77 | 28.8±4.6 | 28.5±4.4 | - | - | 29 | 30 | - | - | - | - | 52.4±10.3 | 51.3±9.3 |

AF= atrial fibrillation; BMI= Body mass index; COPD= chronic obstructive pulmonary disease; CVA= cerebrovascular accident; DM= diabetes mellitus; HP= hypertension; LVEF= left ventricular ejection fraction; MI= myocardial infarction; NR= not reported; PCABG= primary coronary artery bypass grafting; PPCI= prior percutaneous coronary intervention; PVD: peripheral vascular disease; SD= standard deviation.

**Supplementary Table 3.** Demographics of included patients from the selected studies (part 2).

| Study | Prior MI  (%) | | AF  (%) | | COPD  (%) | | Prior CVA  (%) | | PVD  (%) | |
| --- | --- | --- | --- | --- | --- | --- | --- | --- | --- | --- |
|  | PCABG | PPCI | PCABG | PPCI | PCABG | PPCI | PCABG | PPCI | PCABG | PPCI |
| Barakate, 2002 | - | - | - | - | 6 | 7 | 11 | 9 | 11 | 11 |
| Biancari, 2018 | - | - | 8 | 6 | 10 | 10 | 6 | 6 | 22 | 24 |
| Biancari, 2022 | - | - | 8 | 6 | 11 | 11 | 7 | 8 | 21 | 21 |
| Cheng, 2016 | - | - | 9 | 10 | 11 | 7 | 16 | 17 | 9 | 10 |
| Eifert, 2010 | 34 | 54 | - | - | - | - | - | - | 15 | 23 |
| Hakamada, 2021 | 22 | 36 | 3 | 3 | 5 | 4 | 17 | 16 | 11 | 14 |
| Hamiko, 2023 | 18 | 18 | - | - | - | - | 12 | 12 | 16 | 19 |
| Luthra, 2016 | 28 | 69 | 4 | 3 | 12 | 11 | - | - | - | - |
| Mannacio, 2012 | 45 | 48 | - | - | 21 | 27 | - | - | 11 | 13 |
| Massoudy, 2009 | 37 | 60 | - | - | 10 | 9 | - | - | 15 | 18 |
| Mehta, 2012 | 38 | 59 | 4 | 4 | - | - | 7 | 6 | 15 | 14 |
| Miguel, 2019 | 55 | 55 | - | - | 6 | 5 | 5 | 6 | 4 | 10 |
| Nardi, 2022 | 17 | 55 | - | - | - | - | - | - | - | - |
| Niclauss, 2015 | 40 | 44 | - | - | - | - | - | - | - | - |
| O’Neil, 2013 | 36 | 57 | - | - | 7 | 9 | 8 | 7 | 12 | 12 |
| Rai, 2020 | 73 | 75 | - | - | 15 | 13 | 3 | 3 | 16 | 15 |
| Stevens, 2009 | 40 | 50 | - | - | 12 | 13 | - | - | 15 | 15 |
| Thielmann, 2021 | - | - | - | - | 12 | 15 | 10 | 10 | 13 | 17 |
| Velicki, 2013 | 22 | 49 | - | - | 5 | 8 | 5 | 2 | - | - |

AF= atrial fibrillation; BMI= Body mass index; COPD= chronic obstructive pulmonary disease; CVA= cerebrovascular accident; DM= diabetes mellitus; HP= hypertension; LVEF= left ventricular ejection fraction; MI= myocardial infarction; NR= not reported; PCABG= primary coronary artery bypass grafting; PPCI= prior percutaneous coronary intervention; PVD: peripheral vascular disease; SD= standard deviation.

**Supplementary Figure 1.** Leave-one-out analysis for the primary endpoint (perioperative mortality).


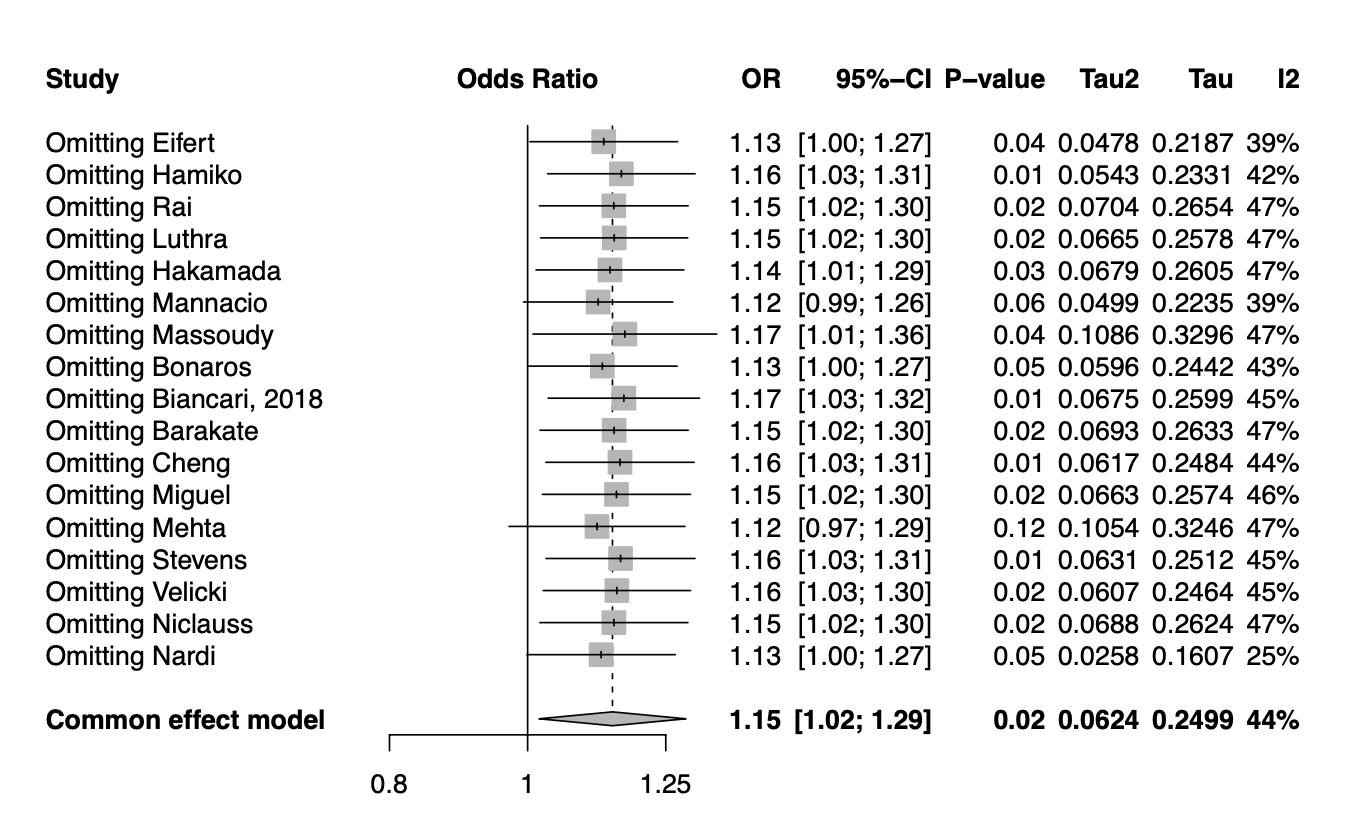


**Supplementary Figure 2.** Funnel plot for the primary endpoint (perioperative mortality).

**Supplementary Figure 3.** Test of proportional hazard assumption (A) and in log–log survival plots (B).

**Supplementary Figure 4.** Forest plot for myocardial infarction.

**
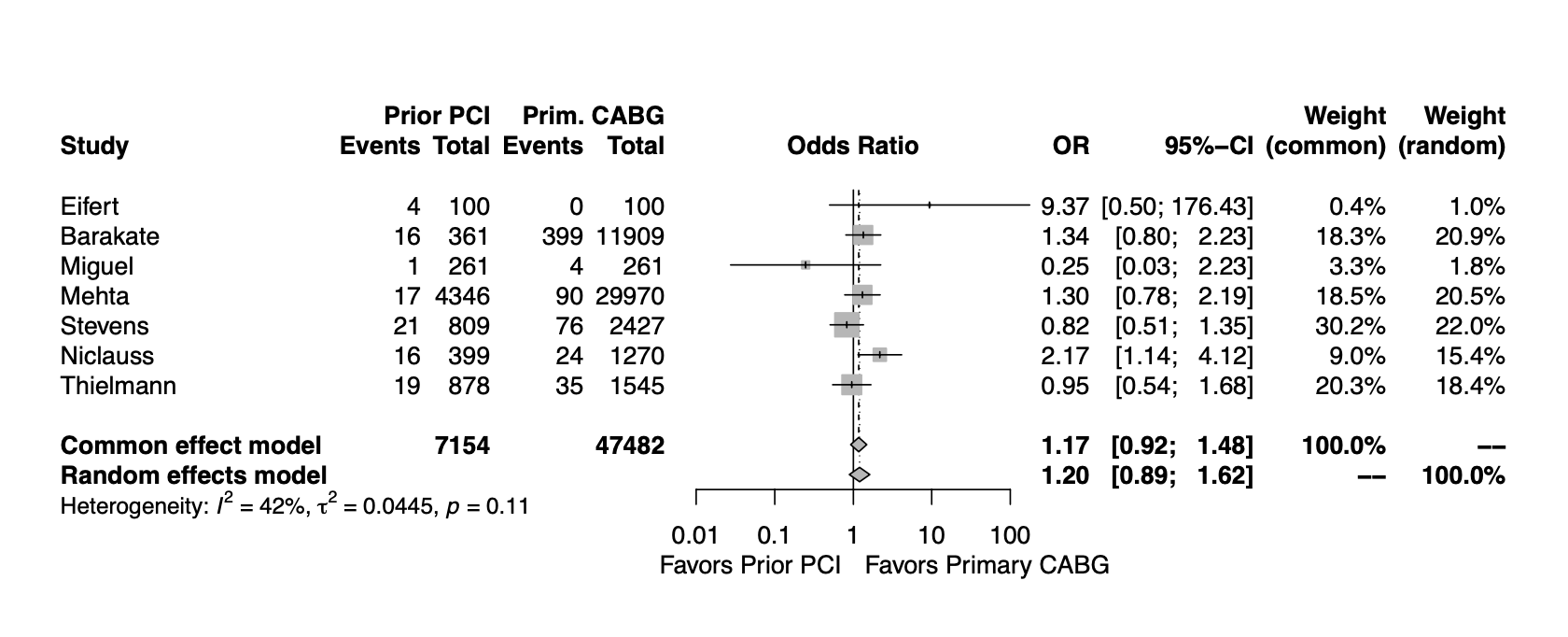
**

**Supplementary Figure 5.** Forest plot for neurological events.

**
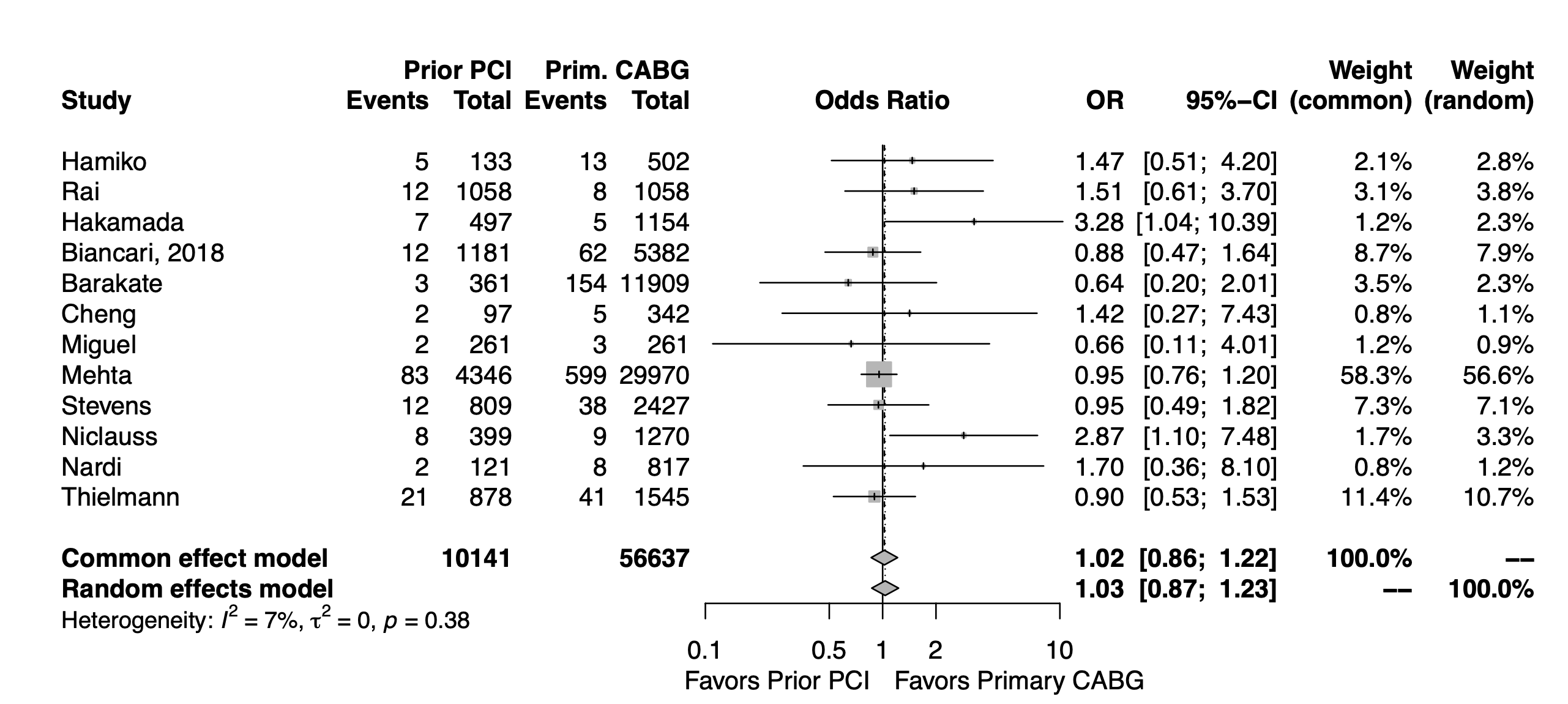
**

**Supplementary Figure 6.** Forest plot for bleeding.

**
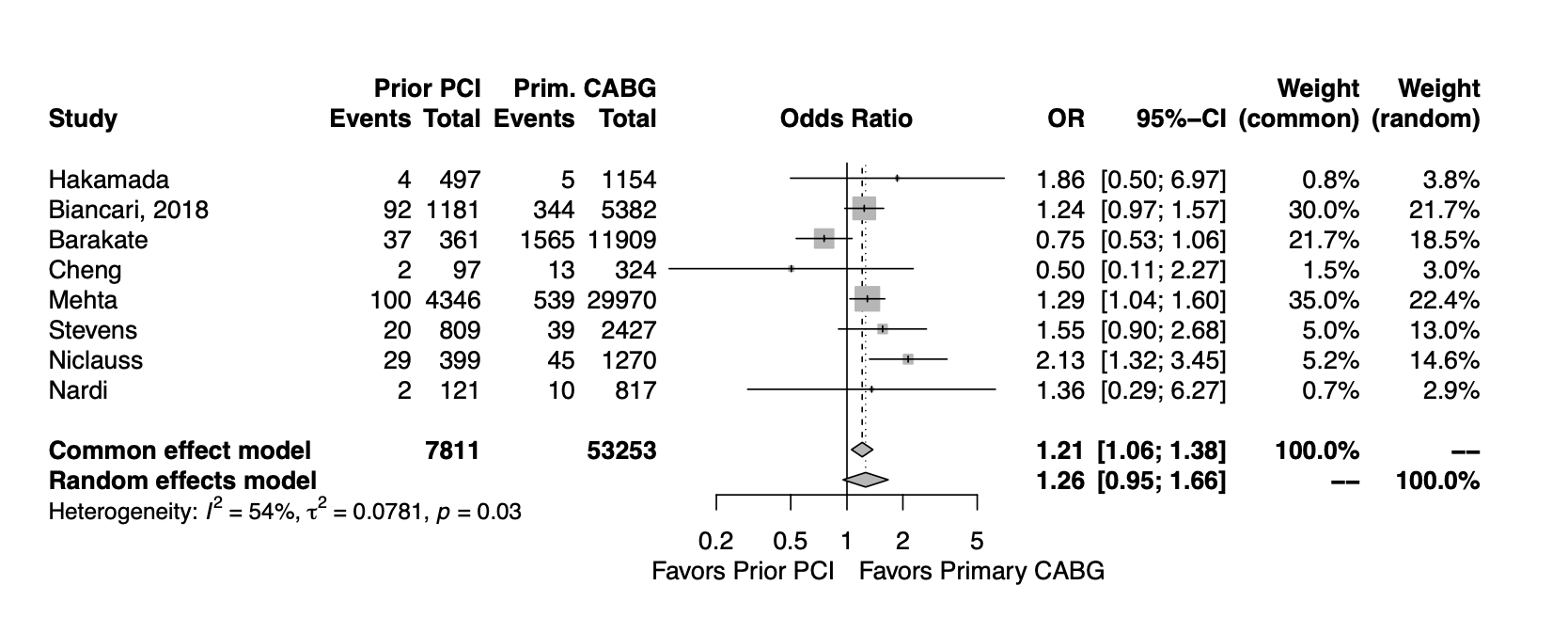
**

**Supplementary Figure 7.** Forest plot for acute renal failure.

**
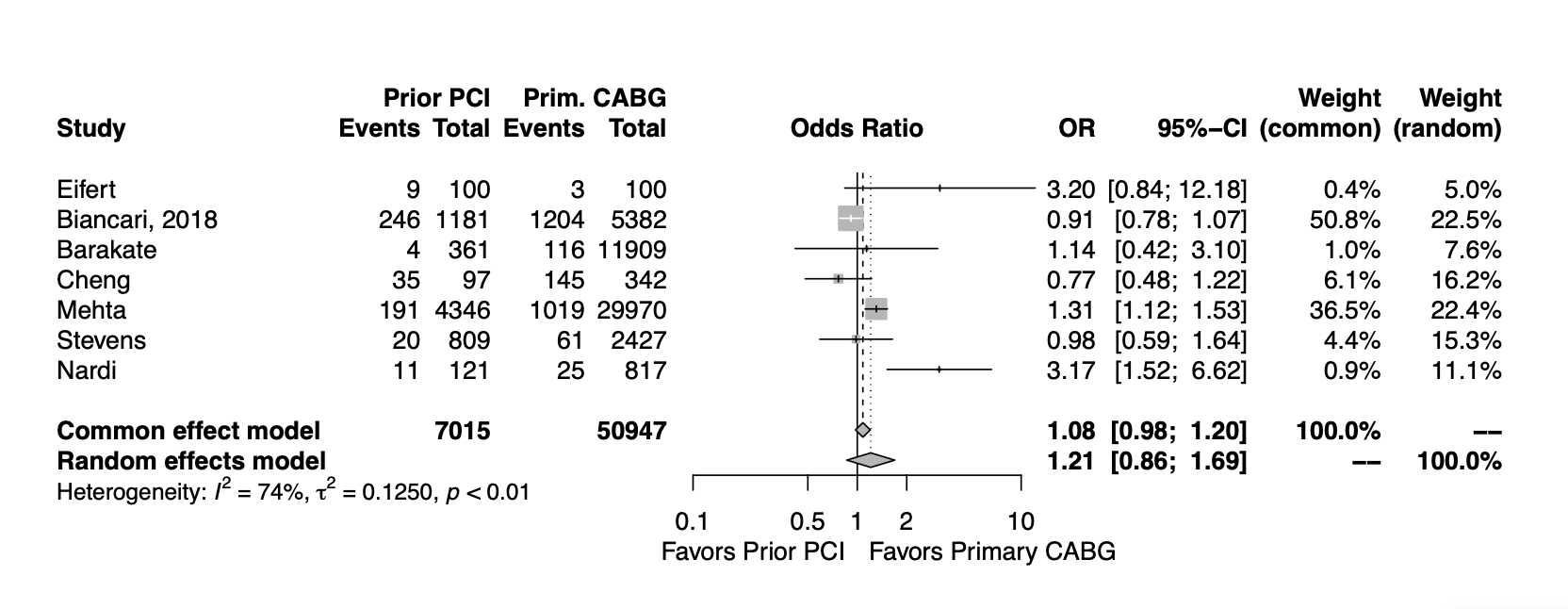
**

**Supplementary Figure 8.** Forest plot for hospital length of stay.

**
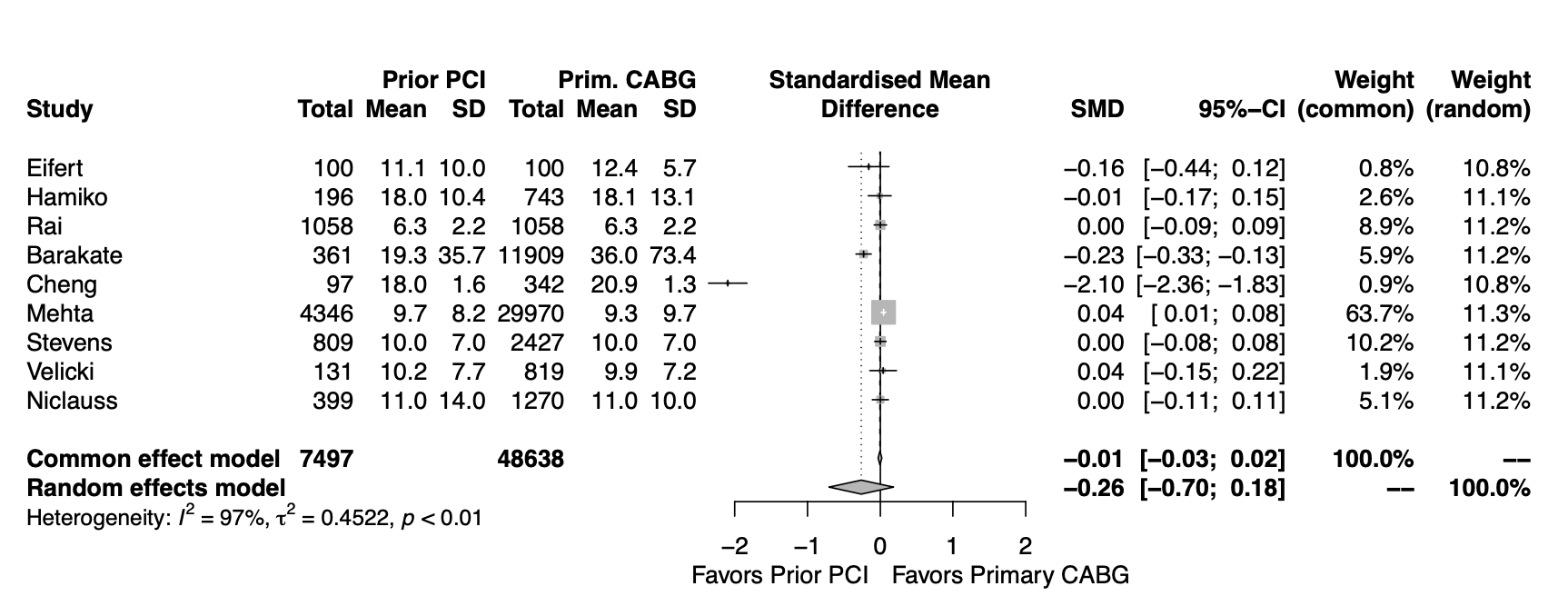
**

**Supplementary References**

1. Barakate MS, Hemli JM, Hughes CF, Bannon PG, Horton MD. Coronary artery bypass grafting (CABG) after initially successful percutaneous transluminal coronary angioplasty (PTCA): a review of 17 years experience. Eur J Cardiothorac Surg. 2003;23(2):179-86.

2. Biancari F, Dalén M, Ruggieri VG, Demal T, Gatti G, Onorati F, et al. Prognostic Impact of Multiple Prior Percutaneous Coronary Interventions in Patients Undergoing Coronary Artery Bypass Grafting. J Am Heart Assoc. 2018;7(20):e010089.

3. Biancari F, Salsano A, Santini F, De Feo M, Dalén M, Zhang Q, et al. Late Myocardial Infarction and Repeat Revascularization after Coronary Artery Bypass Grafting in Patients with Prior Percutaneous Coronary Intervention. J Clin Med. 2022;11(19).

4. Cheng YT, Chen SW, Chang CH, Chu PH, Chen DY, Wu VC, et al. Impact of prior coronary stenting on the outcome of subsequent coronary artery bypass grafting. Biomed J. 2017;40(3):178-84.

5. Eifert S, Mair H, Boulesteix AL, Kilian E, Adamczak M, Reichart B, Lamm P. Mid-term outcomes of patients with PCI prior to CABG in comparison to patients with primary CABG. Vasc Health Risk Manag. 2010;6:495-501.

6. Hakamada K, Sakaguchi G, Marui A, Arai Y, Nagasawa A, Tsumaru S, et al. Effect of Multiple Prior Percutaneous Coronary Interventions on Outcomes After Coronary Artery Bypass Grafting. Circ J. 2021;85(6):850-6.

7. Hamiko M, Konrad N, Lagemann D, Gestrich C, Masseli F, Oezkur M, et al. Follow-Up and Outcome after Coronary Bypass Surgery Preceded by Coronary Stent Implantation. Thorac Cardiovasc Surg. 2023.

8. Luthra S, Leiva Juárez MM, Senanayake E, Luckraz H, Billing JS, Cotton J, Norell MS. Percutaneous Intervention Before Coronary Artery Bypass Surgery Does Not Unfavorably Impact Survival: A Single-Center Propensity-Matched Analysis. Ann Thorac Surg. 2016;102(6):1911-8.

9. Mannacio V, Di Tommaso L, De Amicis V, Lucchetti V, Pepino P, Musumeci F, Vosa C. Previous percutaneous coronary interventions increase mortality and morbidity after coronary surgery. Ann Thorac Surg. 2012;93(6):1956-62.

10. Massoudy P, Thielmann M, Lehmann N, Marr A, Kleikamp G, Maleszka A, et al. Impact of prior percutaneous coronary intervention on the outcome of coronary artery bypass surgery: a multicenter analysis. J Thorac Cardiovasc Surg. 2009;137(4):840-5.

11. Mehta GS, LaPar DJ, Bhamidipati CM, Kern JA, Kron IL, Upchurch GR, Jr., Ailawadi G. Previous percutaneous coronary intervention increases morbidity after coronary artery bypass grafting. Surgery. 2012;152(1):5-11.

12. Miguel GSV, Sousa AG, Silva GS, Colósimo FC, Stolf NAG. Does Prior Percutaneous Coronary Intervention Influence the Outcomes of Coronary Artery Bypass Surgery? Braz J Cardiovasc Surg. 2020;35(1):1-8.

13. Nardi P, Asta, L., Trombetti, D., Bassano, C., Bertoldo, F., Pisano, C.,, Buioni D. FMS, Salvati A.C., Scognamiglio M., Altieri C.,, G. R. Early and mid-term results in patients undergoing primary CABG in comparison with patients with PCI prior to CABG. VESSEL PLUS. 2022;6:1-14.

14. Niclauss L, Colombier S, Prêtre R. Percutaneous coronary interventions prior to coronary artery bypass surgery. J Card Surg. 2015;30(4):313-8.

15. O'Neal WT, Efird JT, Anderson CA, Kindell LC, O'Neal JB, Bruce Ferguson T, et al. The impact of prior percutaneous coronary intervention on long-term survival after coronary artery bypass grafting. Heart Lung Circ. 2013;22(11):940-5.

16. Rai P, Taylor R, Bittar MN. Long-term survival in patients who had CABG with or without prior coronary artery stenting. Open Heart. 2020;7(2).

17. Stevens LM, Khairy P, Agnihotri AK. Coronary artery bypass grafting after recent or remote percutaneous coronary intervention in the Commonwealth of Massachusetts. Circ Cardiovasc Interv. 2010;3(5):460-7.

18. Thielmann M, Wendt D, Slottosch I, Welp H, Schiller W, Tsagakis K, et al. Coronary Artery Bypass Graft Surgery in Patients With Acute Coronary Syndromes After Primary Percutaneous Coronary Intervention: A Current Report From the North-Rhine Westphalia Surgical Myocardial Infarction Registry. J Am Heart Assoc. 2021;10(18):e021182.

19. Velicki L, Cemerlic-Adjic N, Panic G, Jung R, Redzek A, Nicin S. CABG mortality is not influenced by prior PCI in low risk patients. J Card Surg. 2013;28(4):353-8.
